# Correction: Retraction: Heat Shock Protein 27 Is Spatially Distributed in the Human Placenta and Decreased during Labor

**DOI:** 10.1371/journal.pone.0211127

**Published:** 2019-01-16

**Authors:** The PLOS ONE Editors

After this retraction notice [1] was posted, Akrem Abdulsid notified *PLOS ONE* that she had changed her position on the editorial decision in this case. The final paragraph of the retraction notice is hereby updated to the following: AA did not agree with the retraction. KH and FL did not respond.
